# Adult left-ventricular diverticulum and patent ductus arteriosus misdiagnosed as coronary artery disease with infarct aneurysm: a case report

**DOI:** 10.1186/s12872-015-0146-6

**Published:** 2015-11-14

**Authors:** Hong Qu, Tianqi Liu, Haiyan Wang, Dong Wang, Quan Li

**Affiliations:** Department of Cardiovascular Surgery, Shandong Provincial Qianfoshan Hospital, Shandong University, 16766 Jingshi Road, Jinan, 250014 China

**Keywords:** Left-ventricular diverticulum, Patent ductus arteriosus, Coronary artery disease, Angina pectoris

## Abstract

**Background:**

Left-ventricular diverticulum (LD) associated with patent ductus arteriosus (PDA) is extremely rare. We have not found any previous reports of the coexistence of these two malformations. Such an association presenting with chest pain mimicking an infarct aneurysm with angina or a takotsubo cardiomyopathy with chest pain is difficult to differentiate clinically. Here, we discuss several diseases characterized by left-ventricular apical protrusion with chest pain to familiarize clinicians with the differential diagnosis of these diseases.

**Case presentation:**

A 58-year-old woman was referred to our hospital because of complaints of chest pain and dyspnoea, mainly on exertion. An electrocardiograph on admission showed a q-wave in lead I, a Q-wave in lead aVL, and an abnormal T-wave in the limb leads and leads V4 to V6. A transthoracic echocardiograph revealed a PDA and a protrusion arising from the apex of the left ventricle. The diagnosis on admission was PDA and coronary artery disease with infarct aneurysm. To evaluate the source of the chest pain, further evaluations were performed. Coronary angiography showed no abnormal findings. Left ventriculography confirmed the presence of an apical contractile out-pouching. Based on these findings, we revised the diagnosis as LD associated with PDA. The patient underwent transcatheter occlusion of the PDA and was discharged 3 days later. Unexpectedly, transcatheter occlusion resolved the paroxysmal chest pain in this case.

**Conclusion:**

This is the first case report of LD combined with PDA. PDA should be considered in the list of differential diagnosis of chest pain. Several diseases characterized by left-ventricular apical protrusion with chest pain, such as LD, infarct aneurysm and takotsubo cardiomyopathy, can be misdiagnosed as one another. Therefore, it is important to familiarize clinicians with the differential diagnosis of these diseases.

## Background

Left-ventricular diverticulum (LD) is a rare anomaly of out-pouching from the left ventricular wall. In adults, LD can be misdiagnosed as other types of heart disease, such as infarct aneurysm and takotsubo cardiomyopathy [1]. Adult patent ductus arteriosus (PDA) is also rare. In PDA cases, the most common symptoms are dyspnoea and palpitation. It is extremely rare for chest pain to be the main clinical presentation. Here, we reported on an adult LD and PDA case presenting with typical “angina pectoris” relieved by PDA occlusion, which was misdiagnosed as coronary artery disease with infarct aneurysm.

## Case presentation

A 58-year-old woman was referred to our hospital because of complaints of chest pain and dyspnoea, mainly on exertion. The symptoms started at age 18, when she developed progressive dyspnoea and stabbing chest pain located at the cardiac apex, which relieved itself at rest. When she was 35 years old, a PDA was identified with no other cardiac anomaly present. Subsequently, the PDA was surgically ligated through the left chest. The patient led a normal life for 15 years, at which point she started to complain of chest pain and dyspnoea again.

**Fig. 1:**
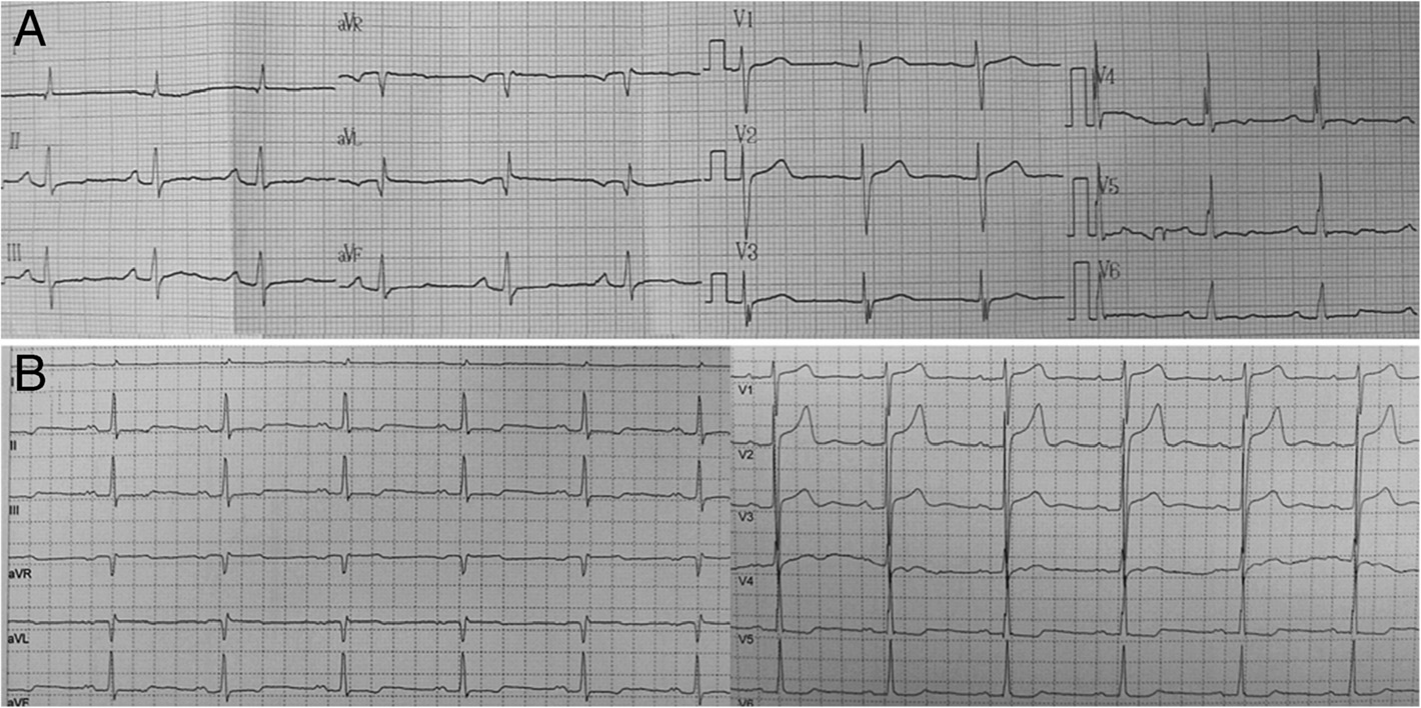
12-lead ECG (**a**). Holter ECG at the onset of symptoms (**b**)

**Fig. 2:**
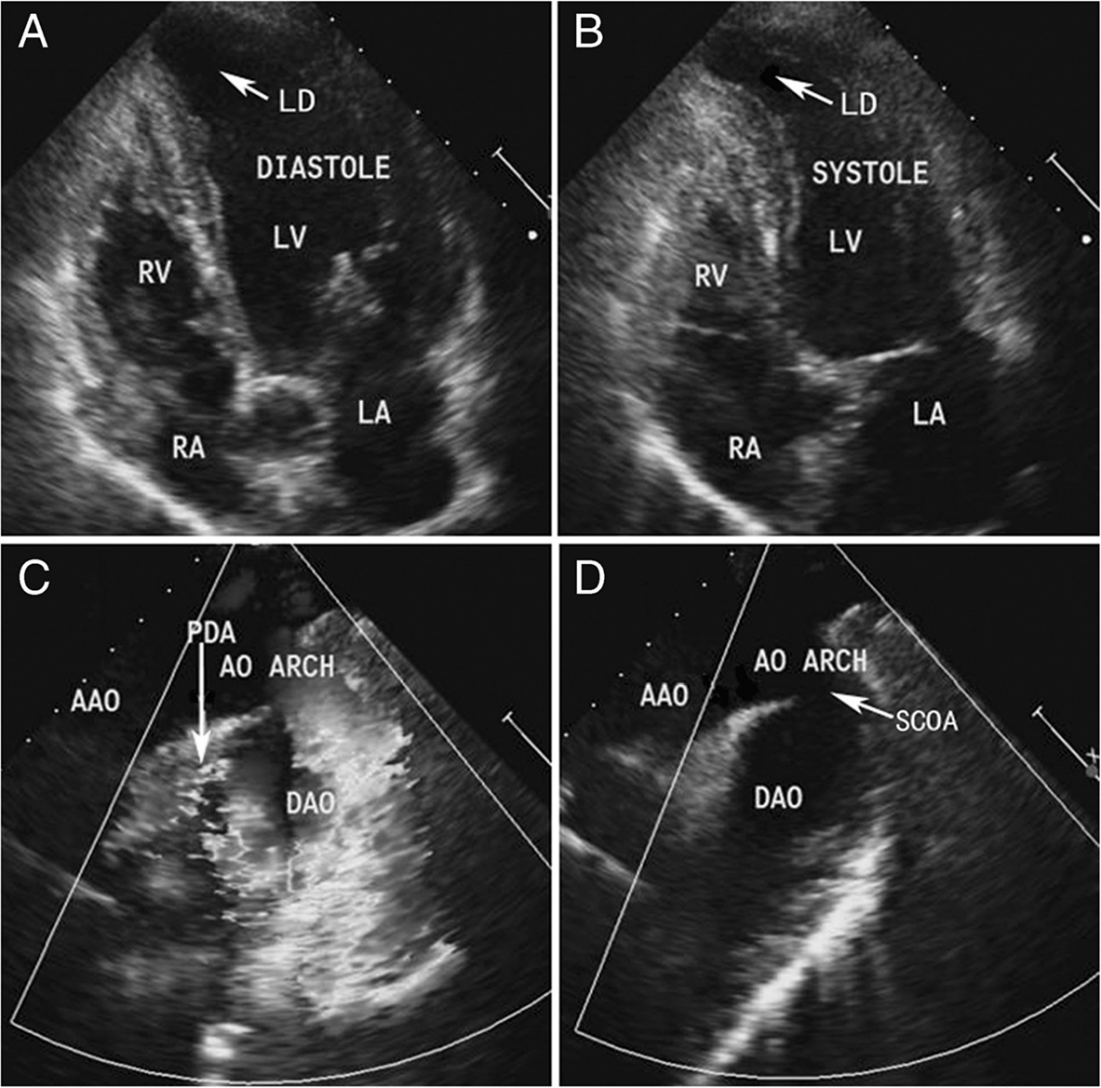
Echocardiogram showing a left-ventricular diverticulum (LD), a PDA and a suspicious coarctation of the aorta (SCOA) (**a** to **d**). LV, left ventricle; RV, right ventricle; LA, left atrium; RA, right atrium; AAO, ascending aorta; AO ARCH, aortic arch; DAO, descending aorta

There was no significant history of psychological stress. Blood pressure was 104/44 mmHg. An electrocardiograph (ECG) on admission showed a q-wave in lead I, a Q-wave in lead aVL, and an abnormal T-wave in the limb leads and leads V4 to V6 (Fig. 1a). A chest x-ray showed cardiomegaly with increased pulmonary vascularity. A transthoracic echocardiograph revealed a suspicious coarctation of the aorta, a PDA with moderate shunt and a contractile protrusion arising from the apex of the left ventricle (Fig. 2a to d). The maximum internal end-diastolic diameters of the muscular protrusion were 2.0 cm × 1.5 cm, and the protrusion beat simultaneously with the enlarged left ventricle with normal ventricular function (ejection fraction 66 %) and wall motion. The pulmonary artery systolic pressure was 41 mmHg estimated by echocardiograph through tricuspid regurgitation velocity. The diagnosis on admission was coronary artery disease with infarct aneurysm, recurrent PDA after surgical ligation and suspicious coarctation of the aorta.

**Fig. 3:**
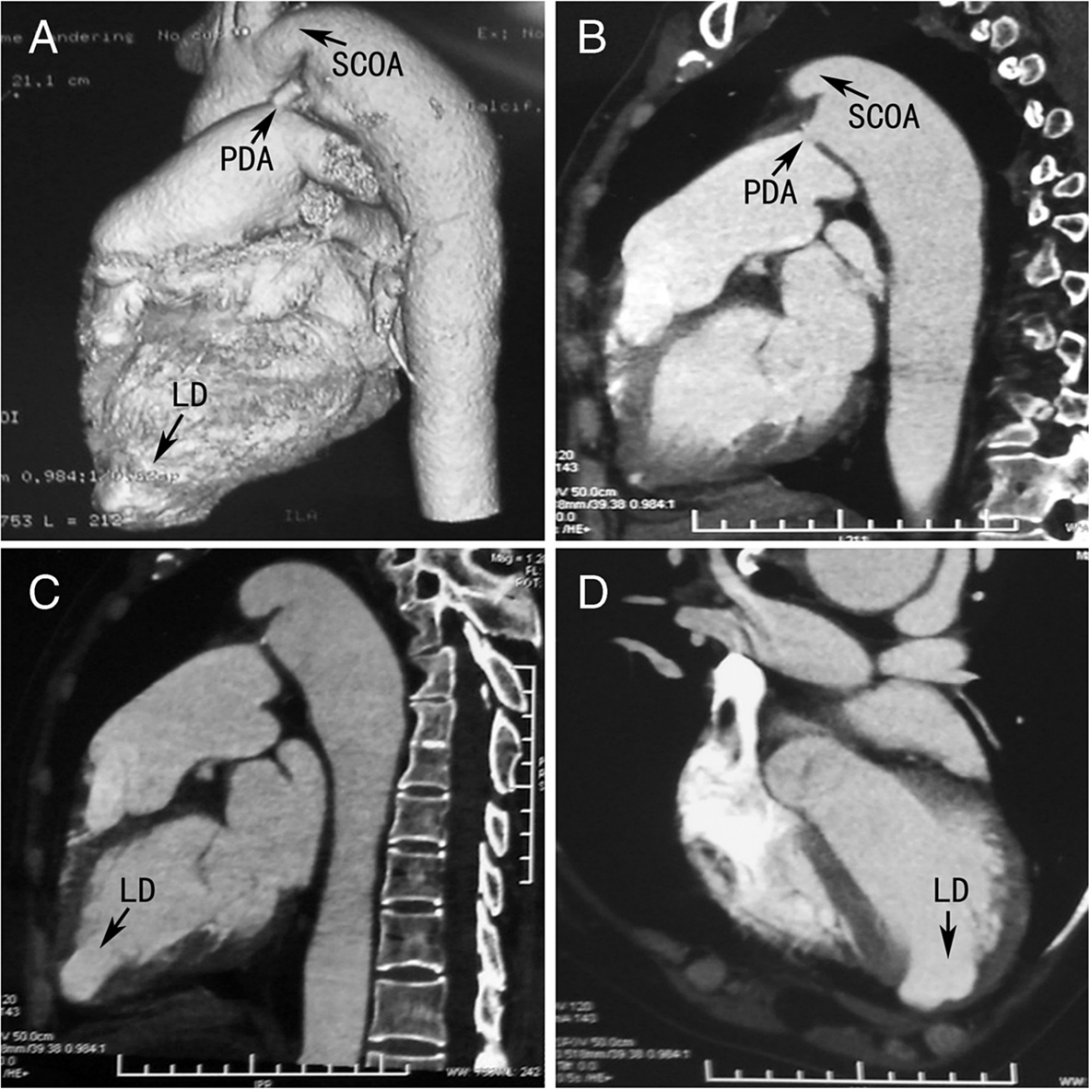
Helical CT showing a PDA, a LD at the cardiac apex and a SCOA of no collateral circulation (**a** to **d**)

**Fig. 4:**
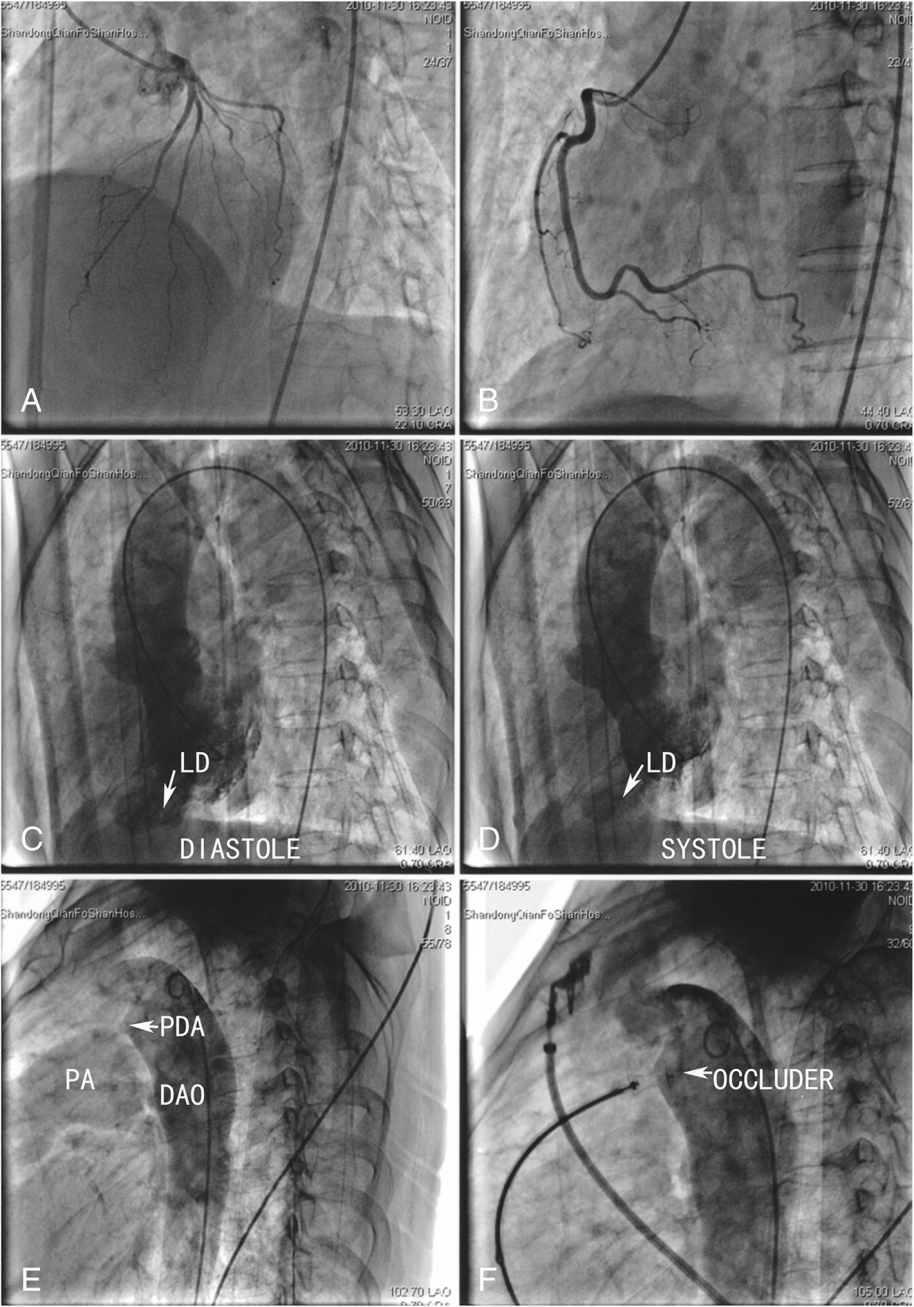
Coronary angiography showing a normal coronary artery (**a** and **b**); Left ventriculography of a left anterior oblique view showing an LD (**c** and **d**) of similar shape compared with the Helical CT (Fig. 3**d**); and an aortic angiogram showing a residual PDA shunt to the pulmonary artery (PA) and good position of the PDA occluder (**e** and **f**)

Further evaluations were performed because of suspicions about the true coarctation of the aorta and coronary artery disease with infarct aneurysm. Cardiac enzyme values were within normal limits. A Holter ECG revealed a normal ST-segment at the onset of symptoms, as it appeared in the routine ECG (Fig. 1b). Helical computed tomography (CT) confirmed the findings by echocardiographic examination. No collateral circulation was detected around the aortic narrowing, and poststenotic dilatation was observed (Fig. 3a to d). Coronary angiography showed no abnormal findings (Fig. 4a and b). Left ventriculography confirmed the presence of an apical contractile out-pouching which was connected directly to the left ventricular cavity by a narrow mouth, and no aneurysmal motion was found (Fig. 4c and d). Retrograde cardiac catheterization through the aortic narrowing demonstrated no pressure gradient. Based on these findings, we revised the diagnosis as LD associated with recurrent PDA after surgical ligation.

The patient underwent transcatheter occlusion, and a 10/8 mm PDA occluder (Starway Medical Technology, Beijing, China) was implanted. An aortic angiogram examination confirmed its proper position and complete closure of the PDA (Fig. 4e and f). After the release of the device, the patient showed significant improvement in symptoms and was discharged 3 days later. At a 12-month follow-up, she was symptom-free.

Muscular LD is generally regarded as a functional accessory chamber. Surgical resection may impair partial ventricular function, and this type of diverticulum is less likely to rupture than the fibrous type. Conservative treatment and anticoagulant are recommended. Therefore, a nonsurgical strategy with careful follow-up was performed in this case.

## Discussion

In the present case, the typical “angina pectoris” and left ventricular apical protrusion are two major factors for the misdiagnosis as coronary artery disease with infarct aneurysm. It is important to familiarize clinicians with the differential diagnosis of diseases with these two factors. The differential diagnosis for chest pain in this patient included LD causing chest pain, severe pulmonary hypertension with angina pectoris, PDA causing chest pain, infarct aneurysm with angina pectoris and takotsubo cardiomyopathy with chest pain.

Muscular LD is very rare in adults and is often complicated by other congenital cardiac or extracardiac malformations. We have not found any previous reports on the coexistence of LD and PDA. Muscular diverticulum is generally found at the apical region and is seldom seen at the basal region [2, 3]. Most left ventricular diverticula are asymptomatic. Only a few reported cases presented with chest pain, and most of them were associated with coronary artery disease. Diverticulum as the cause of chest pain is extremely rare [4, 5]. In this case, the patient was symptom-free after PDA closure, although LD existed. Therefore, LD was not the primary cause of the chest pain.

Angina pectoris related to severe pulmonary hypertension is rarely reported in PDA patients. Because of extrinsic compression of the left main coronary artery, a massively dilated pulmonary artery caused by severe pulmonary hypertension can lead to significant myocardial ischaemia [6, 7]. In the present case, mild pulmonary hypertension (41 mmHg) was detected by echocardiograph and no extrinsic compression of the left main coronary artery was found in coronary angiography (Fig. 4a). Thus, the compression of the coronary artery due to pulmonary hypertension was not the cause of the chest pain.

The cause of the chest pain is not clear. Although much is known about the pathophysiologic mechanisms of coronary steal and left-ventricular overload caused by PDA left-to-right shunt, there are limited clinical data about chest pain as the main clinical presentation. To our knowledge, there have been no more than 2 cases reported worldwide [8, 9]. After the PDA closure, the woman was symptom-free. The fact that chest pain and dyspnoea were resolved first by PDA surgical ligation and later by transcatheter occlusion suggests that the chest pain was directly related to the PDA left-to-right shunt. PDA should be considered in the list of differential diagnosis of chest pain. The mechanism of the chest pain is unclear, but may be due to relatively lower perfusion pressure (diastolic pressure = 44 mmHg) of the coronary arteries before PDA closure or to higher wall stress of the left ventricle during the PDA shunt. Looking the CT scan as well as at the echocardiogram, a significantly dilated and hypertrophied left ventricle and also a slightly dilated and hypertrophied right ventricle can be seen. The low diastolic pressure (perfusion pressure) is the result of the PDA, as a continuous AV (aorta-to-ventricle) fistula leads to lowered “peripheral” resistance. Moreover, the continuous AV fistula also leads to higher left-ventricular pressure. These two factors will significantly reduce the perfusion of the hypertrophied myocardium. One may suggest that under exertion, the heart develops angina on demand. In other words, the hypertrophied ventricle(s) cannot be supplied with sufficient oxygen under the additional burden of low diastolic pressure. This might be even more likely for the diverticulum, which in the coronary angiogram did not show a distinct epicardial vascular supply but rather a tight muscular neck. This might explain the relatively immediate symptom relief after closure of the duct.

Several diseases are characterized by left-ventricular apical protrusion accompanied by chest pain. LD with chest pain in an adult is often difficult to distinguish from left-ventricular infarct aneurysm with angina pectoris. The important diagnostic characteristics of diverticulum, in this patient, were a normal ECG ST-segment, synchronous contractility, normal ventricular wall motion, a normal coronary artery and muscular wall, and a narrow mouth. Takotsubo cardiomyopathy with chest pain should also be considered because the left ventricular shape in the ventriculography looked slightly like a “takotsubo” (Fig. 4c and d). No emotional stress, normal cardiac enzyme values and a normal ECG ST-segment were important diagnostic clues of diverticulum in this case. In a characteristic takotsubo ventricle, the base of the left ventricle is hyperkinetic while the remainder of the left ventricle is akinetic or dyskinetic [10, 11]. However, the echocardiograph of this patient showed normal ventricular function and wall motions, and the apical protrusion was contractile. A Helical CT showed a lobulated protrusion (Fig. 3d). The shape of the protrusion was markedly different from the typical apical ballooning in takotsubo cardiomyopathy.

Some of the important diagnostic characteristics of LD, noted in a previously published review [3], are summarized below and are helpful to exclude post-infarct aneurysm and takotsubo: (1) Examining synchronous contractility, normal ventricular wall motion and normal ejection fraction is one important diagnostic characteristic. Muscular-type LD can contract synchronously with the ventricle and can be identified relatively easily. However, fibrous-type LD cannot contract. Therefore, coronary angiography is an essential tool in these adult cases to exclude post-infarct aneurysm. In a characteristic takotsubo ventricle, the base of the left ventricle is hyperkinetic while the remainder of the left ventricle is akinetic or dyskinetic. However, an LD patient presents with normal ventricular function and wall motions. (2) The varied shapes of the anatomy can also be investigated. LDs of sphere, sphere-like, lobulated, cord, conical and cylindrical shapes are all present. However, these shapes, excluding the sphere or sphere-like shapes, are rare in post-infarct aneurysm and takotsubo patients. (3) Finally, visualizing smaller anatomical sizes can be used to diagnose LD. Although a few reported LDs are large, most LDs are smaller than a post-infarct aneurysm or takotsubo ballooning. Specifically, the mean neck diameter of a LD is only 1.25 ± 0.81 cm. In addition, 83.3 % of LDs are no larger than 2 cm, and 98.3 % are no larger than 3 cm. However, the neck diameters of an infarct aneurysm and takotsubo ballooning are usually larger than 2 to 3 cm.

## Conclusion

We have not found any previous reports on the coexistence of LD and PDA. Our results suggested that PDA can cause chest pain. PDA should be considered in the list of differential diagnosis of chest pain. Several diseases characterized by left-ventricular apical protrusion with chest pain, such as LD, infarct aneurysm and takotsubo cardiomyopathy, can be misdiagnosed as one another. Therefore, it is important to familiarize clinicians with the differential diagnosis of these diseases.

## Consent

Written informed consent was obtained from the patient for publication of this case report and any accompanying images. A copy of the written consent is available for review by the editor of this journal.

## Abbreviations

LD: Left-ventricular diverticulum
PDA: Patent ductus arteriosus
ECG: Electrocardiograph
CT: Computed tomography
SCOA: Suspicious coarctation of the aorta
LV: Left ventricle
RV: Right ventricle
LA: Left atrium
RA: Right atrium
AAO: Ascending aorta
AO ARCH: Aortic arch
DAO: Descending aorta
PA: Pulmonary artery
